# Dimer opening enables brain-type creatine kinase to sense membrane curvature

**DOI:** 10.64898/2026.08.04.742861

**Published:** 2026-09-22

**Authors:** Samantha Gies, Nolan K. McLaughlin, Orianna H. Kou, Ahmed Shubbar, Chen Kong, Tianqi Wu, Sana Khan, Alessandro Ustione, Ian Miller, David W. Piston, Mahmoud Moradi, Michael L. Gross, Wade F. Zeno, Reza Dastvan

## Abstract

Brain-type creatine kinase (CK-BB) buffers local ATP demand through reversible phosphotransfer between ATP and phosphocreatine, yet how this soluble metabolic enzyme engages membrane compartments is unknown. Here, we combine fluorescence microscopy, DEER spectroscopy, hydrogen–deuterium exchange and native mass spectrometry, DEER-and AlphaFold-guided modeling, and long-timescale molecular dynamics to define the pH- and substrate-regulated conformational landscape governing CK-BB membrane association. Acidification promotes curvature-sensitive membrane binding and redistributes endogenous and recombinant CK-BB from diffuse cytosolic pools to punctate vesicular structures and membrane ruffles. Substrates independently promote curvature-sensitive association at neutral pH. DEER and modeling reveal an asymmetric dimer in which the convex surface remains restrained, whereas the concave catalytic–regulatory surface samples pH- and substrate-dependent intermediates. We identify progressive dimer opening as a novel regulatory mechanism whereby acidification and substrate binding increase dynamics across the convex surface and N-terminal dimer interface, generating membrane-competent conformations that facilitate curvature sensing and membrane association. Substrate binding buffers acid-induced deprotection while preserving dynamics near the His191/Ser199 regulatory interface. These findings establish CK-BB as a previously unrecognized curvature-sensitive metabolic enzyme and define dimer dynamics as a molecular switch coupling protonation and substrate occupancy to curved-membrane recognition and localized ATP regeneration, with potential relevance to neurodegeneration and cellular stress.

## Full Text Availability

The license terms selected by the author(s) for this preprint version do not permit archiving in PMC. The full text is available from the preprint server.

